# Examining youth participation in ongoing community and citizen science programs in 3 different out-of-school settings

**DOI:** 10.1080/13504622.2022.2078480

**Published:** 2022-06-16

**Authors:** Maryam Ghadiri Khanaposhtani, Heidi L. Ballard, Julia Lorke, Annie E. Miller, Sasha Pratt-Taweh, Jessie Jennewein, Lucy D. Robinson, Lila Higgins, Rebecca F. Johnson, Alison N. Young, Gregory B. Pauly, Ana I. Benavides Lahnstein

**Affiliations:** a School of Education, University of California, Davis, CA, USA; b Natural History Museum, IPN – Leibniz Institute for Science and Mathematics Education, Kiel, Germany; c California Academy of Sciences, San Francisco, CA, USA; d Natural History Museum, Angela Marmont Centre for UK Biodiversity, London, England; e Natural History Museum of Los Angeles County, Community Science Office and Urban Nature Research Center, Los Angeles, CA, USA

**Keywords:** Informal science education, citizen science, participation, activity theory

## Abstract

We investigated youth participation in three Community and Citizen Science (CCS) programs led by natural history museums in out-of-school settings. Using second generation Activity Theory, we looked at repeated participation over time, collecting and then qualitatively analyzing ethnographic fieldnote observations on focal youth participation and components of the activity systems. We found each program provided multiple and unique access points for youth to participate in environmental science. Further, when facilitators emphasized the scientific goals of the programs clearly and repeatedly, youth participation in the scientific processes of the CCS programs deepened. Access to scientific tools, facilitation in using them, and repeatedly applying them in authentic research, enabled youth to participate in different aspects of CCS, from exploring to submitting biological data. Repeated participation in CCS activities provided the opportunities for youth to try the same type of participation multiple times (intensification), as well as provided the opportunity for youth to try different types of participation (diversification). Our findings suggest that repeated participation in authentic scientific research in CCS contexts fosters youth development of new roles and possible development of environmental science identities.

## Introduction

Informal science learning institutions such as Natural History Museums (NHMs) play a critical role in engaging the public in STEM (Science, Technology, Engineering, and Mathematics) learning (Dierking 2007). Within these institutions, Community and Citizen Science (CCS) programs can engage different audiences, including youth, in authentic scientific research activities (Bonney et al. 2014; Ballard et al. 2017b). We use the term CCS to encompass the range of ways that members of the public can participate in scientific research, which can include varying levels of collaboration with scientists, including citizen science and community science (Ballard et al. 2017b). CCS programs can offer educational opportunities for participants while simultaneously allowing collection of large spatial and/or temporal datasets (Bonney et al. 2009; Shirk et al. 2012; Bonney et al. 2014).

Environmental educators have defined educational goals for CCS participants, including participating in authentic science experiences (Krasny and Bonney 2005; Krasny and Tidball 2009; Phillips et al. 2019), gaining critical thinking skills and science literacy (Bonney et al. 2009), and developing environmental stewardship attitudes and behaviors (Dickinson et al. 2012; Phillips et al. 2019; Stepenuck and Green 2015). Parrish et al. (2019) found that ongoing CCS programs provide opportunities for adult participants to engage in disciplinary practices and skills and to gain an understanding of different aspects of scientific research through repeated engagement with the project. Further, a longitudinal study showed participants value conservation and environmental objectives of CCS programs more when they understand how their work contributes to a greater cause, such as helping scientific research (He et al. 2019). He et al. (2019) also demonstrated that social interactions in which participants support their family or friends can contribute to participants' development of identity and sense of self. All of these benefits rely on repeated participation in CCS over time. While many studies have examined adult participation, few empirical studies have focused on young peoples' participation in environmental CCS, despite youth being integral to the future of science and environmental conservation (Ballard, Dixon, and Harris 2017a; Harris et al. 2020; Harris and Ballard, 2021; Chen et al. 2019; Pitt and Schultz 2018).

Prior empirical educational studies have demonstrated the positive impact of engaging young participants with authentic activities and experiences aligned with scientific practices in real-world contexts. Participation in authentic scientific research can increase participant learning and interest in science (Falk et al. 2016; Harris and Ballard, 2021), increase science literacy, and foster activism (Nasir et al. 2006; Ballard, Dixon, and Harris 2017a; Krasny and Doyle 2002; Schusler and Krasny 2008). Further, Nasir et al. (2006) demonstrated that youth participation in authentic activities and that connecting youth learning to real-life experiences, helped to position youth as knowledgeable and capable. Finally, Ballard, Dixon, and Harris (2017a) showed that three key CCS processes—collecting rigorous data, disseminating scientific findings to real audiences, and investigating complex social-ecological systems—positively impacted youth learning.

### CCS: a pathway to participation in legitimate environmental science research

CCS projects aim to engage participants in a scientific research process in which they have the opportunity to gain skills and knowledge applied in a real-world context (Bonney et al. 2014; Shirk et al. 2012). CCS program participants often have the opportunity to learn and practice science alongside professional scientists whose science, in turn, benefits from the participants' efforts (Newman et al. 2012; Shirk et al. 2012). We begin with the premise that when newcomers are actively and repeatedly engaging in legitimate practices, they may gradually develop specialized content knowledge and gain expertise to contribute to the process (Lave and Wenger 1991). Additionally, participants may become familiar with the practices of a specific community (Wenger 1998), take on the discourses of a community, and be able to contribute to that community (Rogoff et al. 2003). This theory of situated learning (Lave and Wenger 1991) has been applied to understanding adult participation in CCS (Phillips et al. 2019; Jørgensen and Jørgensen 2021; Liberatore et al. 2018), but rarely in the context of youth participation in CCS (e.g. Harris and Ballard 2021), and serves as a useful backdrop for our conceptual framework focused on Cultural Historical Activity Theory (CHAT).

In the case of CCS for young people, participation may be defined as "legitimate peripheral participation", in which young people are participating peripherally in legitimate science practices supported by CCS. The CCS contexts support the immersion in a scientific community and contribute to authentic scientific research through data collection, and/or other relevant scientific practices. Thus, participants who understand their role, the purpose of their participation, and engage with the community in contributing data, may start to identify themselves as becoming part of a scientific community, even if only in a peripheral way (Harris and Ballard, 2021). Over time, whether or not youth are peripheral or central may change as youth identity develops and their learning trajectory advances toward more intensive participation (Lave and Wenger 1991). Young CCS volunteers who may be collecting and submitting data could view themselves as doing a legitimate activity and contributing to science. Thus, it is crucial to understand how the design of CCS settings affects youth participation as well as how young participants position themselves within the scientific practices and within the CCS community. This information can then be used to alter the design settings of future CCS programs and provide opportunities for youth participation and learning outcomes.

Our study of youth in CCS settings builds on previous research demonstrating that aspects of the environmental science learning setting enable or constrain youth participation in science practices (Chen et al. 2019; Harris and Ballard, 2021; Pitt and Schultz 2018; Parrish et al. 2019; Schusler and Krasny 2008). Chen et al. (2019) suggested contextual factors support youth engagement in conservation activities, such as the facilitators' abilities to empower youth by assigning them programmatic roles (i.e. youth ambassador positions). Harris and Ballard (2021) highlighted the role of setting culture, in particular, the role educators play in creating and narrowing forms of participation in science practice and reinforcing them over time. They found that the ways in which participants and educators co-construct the learning environment affects youth participation and science identity development. Pitt and Schultz (2018) studied three citizen science programs engaging secondary school students in collecting ecological monitoring data. Their study showed a connection between program objectives and participants' outcomes and concluded that communicating objectives clearly, positively affected youth understanding of the program, as well as their role and contribution in the program. Here, we investigated three CCS programs led by three different NHMs that engaged youth (5-19 years old) in environmental CCS and asked the following research questions: 1) How do youth participate in NHM-led ongoing biodiversity CCS programs?; 2) How do the constraints and affordances of specific features of the learning environments shape youth participation?; and 3) How does participation change over the course of the program?

## Conceptual framework

### Characterizing CCS learning settings and participation using activity theory

**Figure 1:**
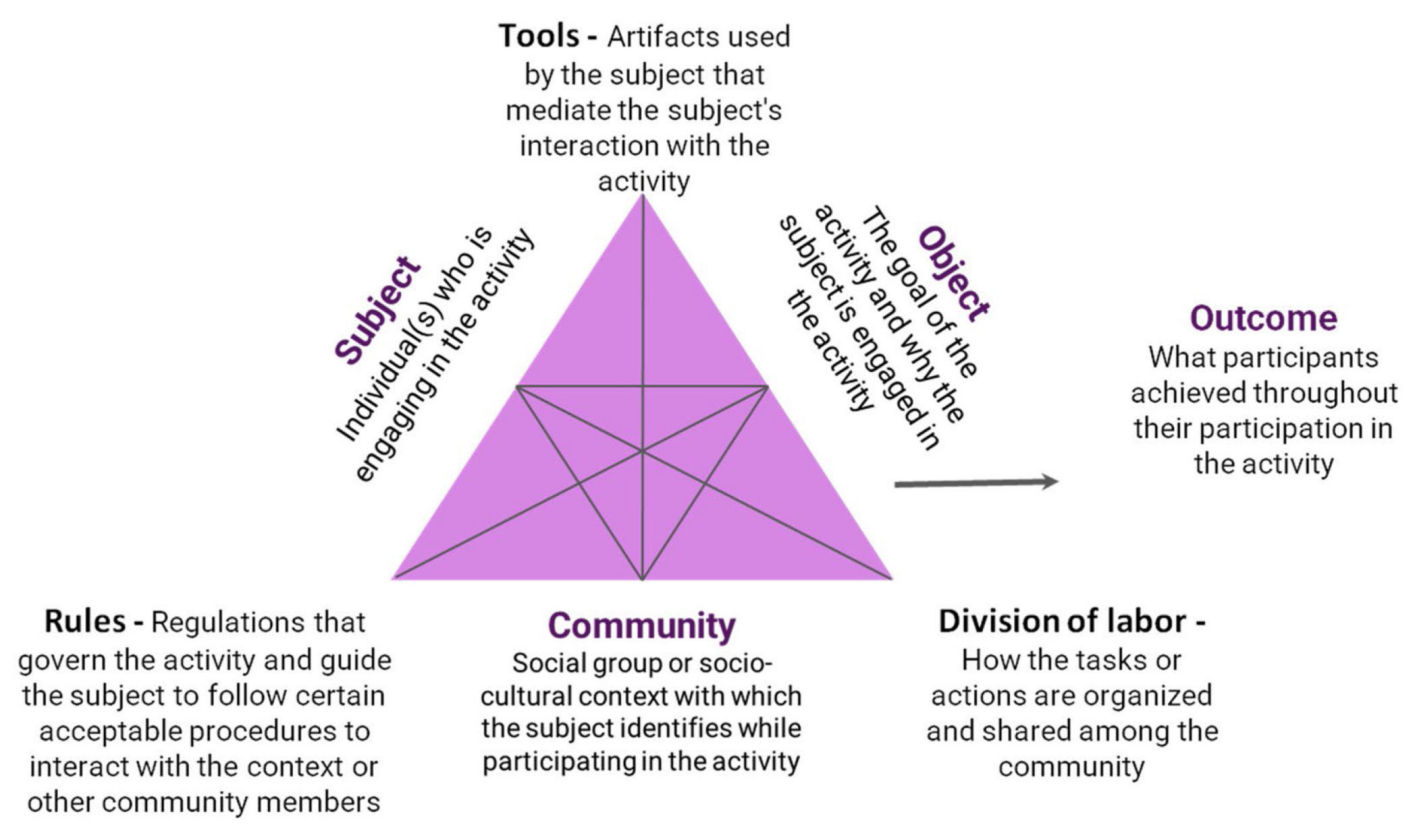
Second generation of activity theory (excerpted from Engestrom 1987).

Building on the notion of CCS participation for youth as situated learning, we developed a conceptual framework that supported our research design, data collection, and analysis focused on the second generation of Cultural Historical Activity Theory (CHAT) to understand the learning environments in which youth participate in CCS activities. Engeström (2000) built on Leontiev's notion of "activity systems" and defined them as objective-oriented, culturally- and collectively-mediated human activity. Individual actions were situated within a context, which constituted the activity system and was considered as the basic unit of analysis (Kuutti 1996). Engeström (2000, 2001) dissected the activity system into six components including Subject, Object, Tools, Community, Division of Labor, and Rules (Figure 1). Each component served a distinct function, but the components were also interconnected with each other and worked together to create an activity. The second-generation CHAT framework helped in the examination of individual participation in learning activities embedded in a social system, whereby a series of small actions work towards a larger goal (Objective) and produce intermediate results along the way (Leontiev 1978). Examining and observing individual behavior was the entryway to investigate youth participation and the structure of the activities (Yamagata-Lynch 2010), that identified how setting features might hinder or foster youth participation and potential learning processes.

In the CCS context, we define the learning environment as bounded by the same six components of the activity system identified by Engeström (1987). We identify each of the broad components of the activity system in each CCS learning context. We speculate that some components may have a significant influence on youth participation, because contextual factors mediate and support young volunteers' participation in order to achieve the "Object" of the activity and realize the "Outcome" (Engeström 1987, 2000). Other scholars have used the lens of CHAT to examine activity systems with respect to participation in environmental education contexts. For example, Lewis and O'Brien (2012) studied elementary school students' participation in monitoring seasonal changes in the Everglades (a national park in the U.S.) and found that a majority of students were engaged in observing, documenting, and collecting data using scientific tools, collaborating with peers, and engaging in more self-directed inquiry. They highlighted that students take on roles according to tasks assigned by their teacher, to achieve goals such as collecting data. Krasny and Roth (2010) suggest that when youth engage in environmental health monitoring, they take on different responsibilities (division of labor), work with community members, and use scientific tools which support their involvement with monitoring and knowledge acquisition that contribute to the larger community. Building on this previous work applying CHAT to similar contexts, we consider individual action and responsibilities as a division of labor and frame it as a type or types of participation. The types of participation are not discrete and exclusive from each other but interconnected. This conceptual framing helped us investigate the relationship between learning settings and repeated youth participation, which could potentially foster learning and identity development.

## Methods

### Overview of the programs

We used a case study design (Yin 2013) to investigate three CCS programs in which youth (5–19 years old) engage over multiple sessions, which we term "ongoing programs" (Ballard et al. 2017b). All programs had "contributory" forms of CCS in which participants collect biological data in a project designed by scientists (Shirk et al. 2012), that may assist scientists, agencies, and non-governmental organizations to address their questions (Harris et al. 2020). Our cases represented three different typical out-of-school youth programming contexts, including after- school (Science Action Club [SAC]), family program (SuperProject [SP]), and environmental education field trips (Big Seaweed Search [BSS]; Table 1). Each program had anywhere from one to hundreds of sites where youth engaged in CCS activities; hence, we selected case study sites for this research primarily based on the advice of program leaders who were implementing the CCS program at the time of study. This was further shaped by participant consent and willingness of parents/guardians and program leaders to facilitate the research.

#### a Data Collection Methods

We observed three CCS programs during four to five sessions each. We observed one SP cohort, one BSS group, and three different SAC sites. To study the repeat participation of youth across three geographically dispersed programs, four observers (one–two per site), were involved in data collection. The number of youth observed in any one session was limited by the number of observers (mostly one per program) as well as the timeframe of each program (SAC sessions were only one hour long). We used stratified purposeful sampling (Patton 2002) to select focal youth representing the gender and age of each program's young people with guidance from the on-site program leader. To capture the repeated nature of ongoing program experiences, we observed each focal youth two to five times resulting in 65 observations in a total of 19 focal youth (nine focal youth in SAC, five in SP, and five in BSS; Table 2).

Observers without a background in qualitative research methods received training in conducting observations and writing fieldnotes. In addition, the team developed observation protocols to ensure that methods were aligned across different settings and observers (Emerson, Fretz, and Shaw 1995). During each session of the programs, one or two observers collected ethnographic fieldnotes, taking an "observer as participant approach" (Creswell 2014). The observers wrote an overall broad setting description for each program, characterizing the components of the activity system at the program level, based on the repeated observations as well as additional information provided by adult facilitators to the whole group, including orientation instructions and "wrap-up" activities at the end of each session. To document youth participation, the observers followed youth for the duration of the observation period to capture youth actions/interactions as well as conversations with others. The observers summarized all CHAT components in a separate table within youth participation fieldnotes. Later, the observers transcribed all the fieldnotes for both program broad setting description and youth participation as well as their reflective comments. Due to the different program designs, the observers could observe multiple participants in SAC and BSS, but for SP, only one youth could be observed per session. To maximize the number of youth observations in SAC and BSS, ethnographic field observations lasted 20 min for each focal youth. For SP, the researcher was able to have longer observation intervals of up to 60 min. While we may have missed youth participation in the CCS activities occurring outside of our observation intervals, our methods struck a balance between observing multiple young people in a program and depth of observation of any individual.

This study has been approved by the Institutional Review Board (IRB) at the University of California, Davis, USA (# 624197-13) and Human Research Ethics Committee (HREC-2726- Herodotou) at the Open University, UK.

#### b Data Analysis Methods

Analysis of the data included a series of iterative stages of interpretation that allowed us to be reflexive between our initial theoretical frames and themes emerging from the data (Patton 2005). Our unit of analysis is a CCS activity that is objective-oriented containing multiple actions, has groups of conditioned operations in which youth take on different responsibilities to achieve the outcome (Roth, Lee, and Hsu 2009), and is taken by individual participants. For example, in an activity of exploring the beach to find seaweed, a youth could take multiple actions including exploring the beach, observing seaweed closely to see the different features, using the guide to identify the seaweed, and communicating his finding with his peers.

As the **first step** of the analysis, observers wrote an *analytical memo* to describe the participation in the CCS activities (where applicable) of focal youths over time. Guiding questions were provided to support observers in extracting relevant information from their fieldnotes, for example: What does youth participation look like in each session of the CCS program? How does youth participation change over the course of multiple sessions? What setting features are playing roles? What evidence can back up our interpretations?

For the **second stage** of analysis, written profiles were developed for the 19 young people who participated two or more times as we were interested in repeated participation (*n* = 19; 65 observations). We used the ethnographic fieldnotes from their participation over time (Ballard, Dixon, and Harris 2017a), the analytical memos, and broad setting descriptions in order to focus on the ongoing nature of these programs and participants' repeated experiences with the learning settings. The process of profile writing for each focal youth was iterative and involved two researchers' corroboration on each profile, ensuring an observer who attended the event, and an additional person confirmed the analysis. Each profile consisted of:

- An overview of the participant including demographic information and the chronological summary of their participation in the program over time.
- Key "action and/or interaction episodes" in which youth engage in CCS-related activities and/or interactions and conversations with others. Each profile had at least one episode or a sequential compilation of all the episodes when the individual engaged in science, environment, and nature-related activities. Using "episodes'' as a unit of analysis, we took an inductive yet systematic approach, including direct quotes from the fieldnotes as "evidence vignettes'', followed by previously categorized type(s) of participation, following Lorke et al. (2021), that a youth might engage in during CCS programs based on biological recording (Table 3). Lorke et al. (2021) identified these types of participation specifically in BioBlitz settings. These are CCS events aiming to generate a biodiversity inventory in the form of biological records in one particular location over a short time frame (usually 2–24 h). We consider the observable demonstrations of youth engaging in these types of participation as engaging with science practices (Ballard, Dixon, and Harris 2017a). In addition to our observations, we looked for evidence of Recording in program level, through 1) checking the iNaturalist account of the youth/their parents or program-run accounts in SAC and SP for data submission; 2) checking whether the data recording sheets for BSS were submitted to the museum. This evidence was followed by a claim about the impact of any components of the activity system on youth participation.
- A summary of cumulative participation

A *Changes over time* document was written according to the analytical memo and the episodes in each observation to look at individuals over the course of several CCS program sessions (Berkes & Folke 2002; Engeström 1987, 2001; Folke et al. 2002). Researchers examined whether repeated experiences with the program led to *intensification* of the same type(s) of participation over time (more frequency counts), which Lave and Wenger (1991) call moving from peripheral to central participation and related to the development of role and identity. In addition, multiple engagements with CCS could lead to *diversification* of experiences, i.e. engagement in different types of participation in CCS, which Lave and Wenger (1991) describe as a transition from peripheral to full participation. Then, we looked for any patterns in these changes in participation in order to cluster the youth into categories.

**Figure 2:**
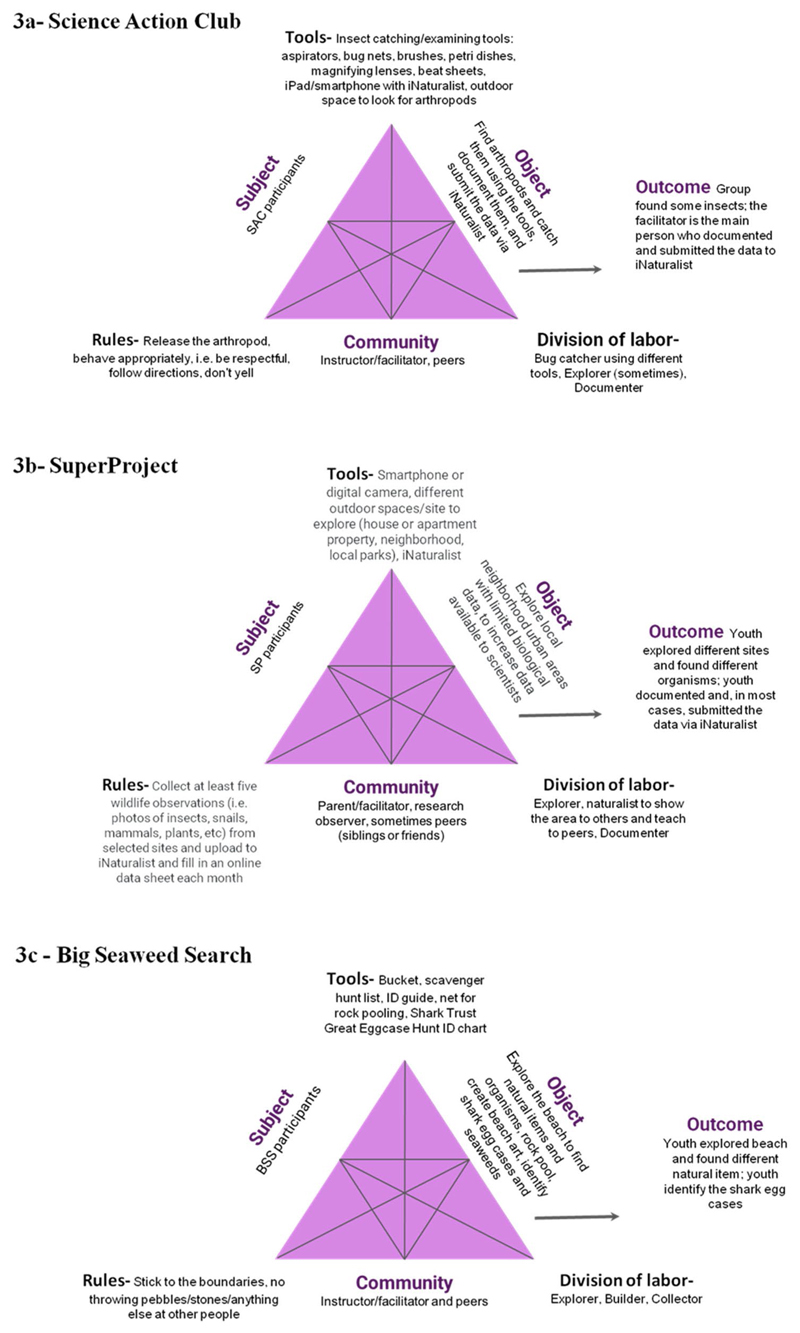
(a, b, and c). The components of an activity system for each program SAC, SP, and BSS, respectively, showing how participants' actions and performance are mediated by tools within a social and environmental context, using Engeström (1987).

The **third stage** of analysis involved the application of Engeström's (1987) second generation activity theory to analyze interactions between the six components of the activity system to understand the role of each component and how these impacted the focal youth/Subject, and whether the youth achieved the objective of the activity ("outcomes" in Engeström 1987; Figure 2). For each program, we did this at the individual level (experiences of each focal youth) and at the collective level (groups of individuals as the Subject in the activity system), in order to describe and isolate the setting features of each CCS learning environment that might be influencing youth participation (Table 4).

The last stage of individual-level analysis involved one researcher (the first author) thematically coding (Patton 2005) all episodes within each profile according to the types of participation and CHAT components broadly defined above using Dedoose (Version 8.0.35 2018). We then queried each of the types of participation and their relative predominance and/or rarity. We created a co-occurrence matrix table and examined the relationships between all of the types of participation and the CHAT components. This symmetric, code-by-code matrix presented the frequencies for which all code pairings were applied to the same episodes and, by default, overlapping excerpts.

##### Collective-level analysis

We used the coded individual-level profiles and the "broad setting descriptions" to query each of the elements of the activity system to identify and analyze said systems at the program level for each of the three programs. One researcher (first author) interpreted and qualitatively coded excerpts as evidence of the change over time to identify the main CHAT components that were emerging in the experiences of youth who showed changes in participation over time (Patton 2005).

## Findings

### Youth participation in ongoing CCS programs

Of 19 focal youth, 15 were observed engaging in one or more Types of Participation in CCS. We present evidence of each type and examples from fieldnote observations alongside focal youth pseudonyms, age category, and gender (Table 5). We note that for four of the focal youth we did not see evidence of engagement in CCS activities, rather they engaged in other activities such as building a sandcastle, playing tag, or nature-based crafts. While those activities may have been potentially meaningful for youth in other ways, for the purposes of this paper, we don't further discuss these non-CCS activities.

Overall, we found that *Exploring* and *Observing* were the most common types of participation in these ongoing programs. *Exploring* and *Observing* occurred in all three programs. The other three types of participation (*Identifying, Documenting,* and *Recording)* were primarily observed in SAC and SP (Table 5). Within the category of *Identifying* organisms, we further refined our definitions to separate the two main ways through which youth achieved an identification; either by using their prior knowledge and/or according to certain features of the specimen, predominantly in SAC, or by using the iNaturalist smartphone app (predominantly in SP). *Recording* was a common type of participation in SP and rare in other programs. It was undertaken by youth independently, in collaboration with adults, or solely by adults without any youth involvement. In SAC, data were *Documented* and *Recorded* primarily by facilitators, with or without youth involvement, while in SP all youth themselves experienced *Documenting* and often *Recording* (Table 6).

### Characterizing the relationships between the various components of the activity system and youth participation

Our second research question asked how the specific features of the learning environments shaped youth participation in CCS. Youth in all the programs were engaged in nature exploration and participated in CCS to different degrees. The type of participation and degree of engagement in the activity differed depending on a number of features of the learning environment including the Objective of the activity, the Tools available, and the context of the learning environment. We found the different components of each activity system varied in the extent to which they influenced youth participation (Figure 2).

### Relating the type of participation to contextual factors

The presence of particular components of the activity system and the quality of these components influenced youth participation by supporting or hindering the opportunities for and nature of that participation. The Objective of the program shaped to what extent each of the other components influenced participation, as it steered the scope and focus of the whole activity. For more detailed analysis about how each type of participation is paired with each of the components of the activity system see Appendix A. For example, Community and Tools were found to be associated with Exploration/Discovery in many more episodes. We next provide descriptive examples of how the Objective, Community, Tools, and Division of Labor components of the activity system seemed to influence youth participation in the CCS programs.

#### The influence of the Objectives on youth participation

Our findings showed that all programs were broadly designed as CCS programs, and programs with less clear CCS objectives had few opportunities for youth CCS-related type of participation. In addition, the objective of all the available activities within the programs was important and affected youth participation whether the objectives were aligned with CCS or not. For example in SAC and BSS, some of the available activities had explicit CCS-focused objectives that resulted in collecting biological data. For these programs, the majority of activities were more oriented towards environmental education or building general scientific skills (e.g. making insect models with marshmallows or making boats out of natural items). In SAC, some but not all "Bug Safari" sessions were framed by the on-site educator as having the objective to collect observations about insects for scientific research. The SP program explicitly framed the CCS objective to engage participants to make nature observations using smartphones in their own neighborhoods with the goal of understanding nature in the Los Angeles area to inform NHM researchers, and this was reinforced in the training sessions. Our observational data showed that SP youth were continually engaged in CCS activities, visiting different locations (backyard, neighborhood, and local parks) to Document species occurrence records (photos) using iNaturalist. In BSS, we observed that CCS activities sat within a wider program with the main objective being environmental education, Exploration and connecting youth with nature. While we observed youth engage in a variety of environmental education activities, only two activities had the clear objective of youth contributing to science, and for those we observed youth participating in CCS surveys for shark egg cases and seaweeds. Overall across SAC and BSS, our observational fieldnotes revealed some inconsistency in the clarity of objectives shared with youth during each program session and a lack of connecting individual youth actions to the broader CCS objective.

#### The influence of Tools on youth participation

SAC participants had access to a variety of scientific tools that seemed to support their Exploring and Observing activities in the outdoor settings of the afterschool program. Access to the tools for Documenting and Recording (iPad or smartphone) was more limited and mainly handled by facilitators, and youth were occasionally involved. In SP, all participants had access to smartphones or tablets (their own or their parents') with the iNaturalist app or camera, which made it possible for them to directly engage in Documenting and Recording, and almost all submitted their own scientific data. In BSS, youth had easy access to basic tools (such as buckets or natural items) associated with the nature-play focused activities, to build things such as boats or shell necklaces. However, because the CCS program was specifically designed to require no special equipment, there was limited access to scientific tools, only ID guides and data sheets, to support youth engagement in CCS-focused activities. This limited access to Tools, in turn, limited the types of CCS participation youth exhibited. Regardless of the age, youth who had access to scientific tools and received support from adults, participated in a variety of CCS-related types of participation.

#### The influence of Community on youth participation

Our data showed that each CCS program provided a variety of opportunities for youth to interact with different people who formed their CCS Community. In addition to the research observer, SAC and BSS participants had access to their peers and facilitators, whereas SP participants had access to their parent(s), sometimes friends and siblings, and occasionally museum scientists in NHMLAC meet-up sessions. Our data showed access to and support from the Community provided opportunities for youth to communicate about their observations, share their prior knowledge of the area, identify species through discussion about organism traits, and receive or provide feedback.

#### The influence of Division of labor on youth participation

Each program allowed for a variety of roles and different distribution of labor across the community, and this provided multiple opportunities for youth to try and practice different roles. Across all programs, almost all youth took on roles as "observer" and/or "explorer." SAC specifically engaged youth in Exploring multiple times applying different exploration tools. SP, with a strong CCS-oriented objective, provided a range of ways that youth could participate and take on a variety of roles, with an emphasis on Documenting, so that the division of labor was more widely distributed across the community. All SP participants took the role of "documenter" and "identifier," perhaps due to the environmental surveying focus of the programs and access to smartphones with iNaturalist app. BSS had limited opportunities for participation in CCS activities and youth were engaged in finding nature to make art. The quality of the division of labor could be connected to the CCS objective of the program and the corresponding activities that could hinder or support youth trying different CCS-related types of participation.

### Changes in youth participation over the course of the program

Young people's participation changed over the course of their engagement in the programs. We found evidence of *intensification* (multiple episodes of the same type of participation over time) and *diversification* of their activities (trying different types of participation over time), depending on the program and for different focal youth. Our analysis of the ethnographic profiles revealed that eight out of 19 showed changes in their participation over time.

For youth who showed change in participation over time, most engaged in four or more types of participation in CCS (six out of eight youth). Each of these eight focal youths had access to tools to engage in science practices and were supported in using them by others in the community (instructor, parents, peers). All eight youth were part of SP or SAC, in which there were more opportunities for youth to participate in CCS-oriented activities, compared to BSS where the program focus was heavily weighted towards non-CCS environmental education activities. In addition, most of the SAC participants were observed twice while SP participants were observed three to four times showing the SAC participants had less exposure to CCS activities. In SAC and SP, we observed five sessions of each program, and participants who participated in more sessions outside of our observations may have gained more experiences and tried more CCS-related types of participation. When we looked for patterns in the ways those eight focal youth's participation changed over time, we identified three clusters/categories of change: a) becoming competent *explorers* and *observers,* b) gaining *mastery of disciplinary science practices,* and c) becoming a *naturalist* to teach others. The categories were not mutually exclusive. This illustrates different ways that participation can intensify, and how different components of the activity system interact, in particular the ways in which the participant (Subject) uses scientific tools to achieve the objective repeatedly. This may indicate the impact of deepening the experience by repeating the same type of participation.

In the first category, becoming competent *explorers* and *observers,* three participants became actively involved and competent in Exploring different locations, Observing specimens and their behavior closely, and Documenting them. For example, Scott (High school-aged male participant, SP) at first was passively participating, accompanying his mother who was highly engaged in biodiversity monitoring through iNaturalist in their neighborhood. By the second observation, he became actively involved in Exploration of the neighborhood, Observing organisms, and pointing them out to his mom and encouraging her to Document them. By the third observation, Scott actively began Exploring and investigating new natural features (e.g. leaf patterns), Observing organisms closely (e.g. a spider, a mockingbird, and ants next to tree sap), and Documenting. In the fourth observation, when his friend joined him and his mom in the neighborhood survey, Scott took the initiative to communicate to his friend various things related to nature and the objective of their participation in SP.

For the second category, gaining *mastery of disciplinary science practice,* the five focal youth who displayed this used particular scientific tools repeatedly over the course of the program. They learned how to use them from the facilitators, and developed science skill mastery and ownership in using tools over time. They all initially showed little interest in using the tools provided, but over the course of the program sessions began using the tools with increasing frequency and engaged in more diverse types of participation. Access to scientific tools offered opportunities to frequently engage in science practices. For example, Joey, (Middle school-aged male participant, SAC) in the first observation was not engaged in the "Bug Safari" activities at all, but he became interested in using the bug net to Explore the area and catch insects over the course of the program. After several attempts, he made fewer failed attempts and captured more challenging insects (e.g. a fast-moving bee) and later helped the facilitator fix another participant's broken bug net. Another example was Ashley (High school-aged female participant, SP), who gained knowledge and experience in using a tool over the course of two sessions. While she began the program engaging in Exploring, by the fourth observation, Ashley had integrated iNaturalist into her everyday life by Documenting and taking photos of organisms she found. Throughout her day, Ashley said she felt recognized by others (her family and her school friends) as the person who used iNaturalist, and was asked at school to Record and Identify species using the app.

The third category, *becoming a naturalist,* included two young participants who visited different outdoor locations multiple times and became familiar with their different local environments; both of these were participants in SP. They were able to communicate their knowledge to their Community (giving feedback to peers, siblings, or parents), Identify certain organisms, compare different sites and their biodiversity, and teach others how to use scientific tools. For example, in the first and second observations, Charlie (High school-aged male participant, SP) was passively participating and sometimes helping his mom Documenting. By the third observation, he was communicating his prior knowledge of different species in the area with the research observer. He became skillful in using tools and Documenting, for instance by trying to take multiple photos per observation. Charlie recognized that his neighborhood survey resulted in fewer wildlife observations in comparison to his backyard, explaining that 'the neighborhood survey is the hardest because I don't tend to find a lot.' In the fourth and fifth observations, he took on different roles engaging in Exploring, assisting his mom to find and Document organisms, and mentoring another youth. In one visit to a park with another SP participant (Laurence, Elementary male youth), Charlie took on a mentorship role, communicating with Laurence, sharing some of his skills on how to use certain tools as well as his knowledge about wasps and their parasitism behavior.

## Discussion

### Youth participation within and across the ongoing environmental monitoring programs

Our results showed the unique context of each program, which gave youth opportunities to participate in CCS through a variety of entry points. At the individual level, efforts to understand different types of participation and changing forms of participation are important because they shift the focus of educational research from solely considering knowledge acquisition to considering how learning environments afford different forms of participation that can expand into identity work, agency, and how participants see themselves performing (Boyer and Roth 2006; Fenwick 2006). Our study suggests that when programs provide a variety of social and material resources to support individuals' participation, these conditions thereby create multiple opportunities for youth to participate and position them to engage in different disciplinary science practices (Boyer and Roth 2006).

While all programs had the potential to engage youth in all steps of biological research as it relates to biodiversity-focused CCS programs (Exploring through Recording), not all participants experienced all types of participation. Fewer youth engaged in Documenting and Recording, which are important steps to submit biological data in contributory CCS programs, and these steps could be considered to differentiate environmental education from CCS, a point also raised by Lorke et al. (2021) in the context of BioBlitzes. However, engaging in some but not all aspects of the scientific process used for biodiversity-focused CCS (i.e. Exploring through Documenting) is still important both for learning and for contributing to biodiversity research. Partial engagement may influence a participant's identity with science, as reported by He et al. (2019) who found that participants reported strong identity development as data collectors, even if they avoided other parts of the scientific process such as data analysis. Further, Krasny and Roth (2010) suggested that participation in a diversity of learning experiences improves adaptive capacity among individual participants, in turn leading to building a more resilient system at the collective level.

By principle, environmental education and science education have a 'mutualistic' relationship (Gough 2002); they complement each other. Socio-scientific issues, environmental science literacy, and climate change education fields are recent examples of the synergy between environmental education and science education (Dillon 2014). However, conceptualizing the environment as an "object of study" and embedding environmental education in disciplinary agendas of science education are some of the aspects that have contributed to a long-standing tension between these fields (Palmer 1998). Environmental or conservation citizen science projects can bring both fields together (Wals et al. 2014) by centering participation, community engagement, and ecological citizenship with science. This study reflects this mutualism between science and environmental education by highlighting the importance of participation in science practices while specifically grounding youth meaning-making in environmental topics about urban biodiversity and climate change.

### Constraints and affordances of CCS settings through the lens of the second generation of CHAT

By examining the three CCS programs through the lens of the second generation of CHAT, we were able to identify how the components of the activity systems were linked, how youth interacted with them, and what actions were being taken to achieve the objective (Krasny and Roth 2010). This research explores types of participation in ongoing CCS programs. This work builds upon the 'Type of Participation Framework' developed by Lorke et al. (2021) where participation was examined in short term CCS events.

#### Objective

Although the broader objective of all three activity systems was "submitting biological data", each program framed its CCS objectives differently, and particular sessions within the programs didn't always align with the broader objective of the overall program. This variation can influence whether and how CCS participants understand the objectives of each activity and how their actions contribute to the overarching objective of submitting biological data. It also could impact development of identity with science (Roth, Lee, and Hsu 2009; Haywood, Parrish, and Dolliver 2016). SP and, to some extent, SAC participants, took part in more diverse and intensive CCS activities in relevant and significant ways toward achieving the collective CCS objectives. On the other hand, BSS participants had limited opportunities for participation in CCS because the program objective focused primarily on environmental education in the out-of-doors (Boyer and Roth 2006). How program leaders frame the program and the objective of the activity matters, and also how they link the goal of each action to the broader objective. Framing program activities explicitly as CCS affects youth engagement and efficacy in trying different roles (Lewis and O'Brien 2012; Pitt and Schultz 2018). The stated Objective of the programs also influences the participants' perspectives about the objective (Fenwick 2006). We found both considerations affect the diversity and intensity of young people's participation, and thereby opportunities to build the skills needed to engage with their learning environment (Fazey et al. 2007; Chawla 2008; Roth, Lee, and Hsu 2009; Krasny and Roth 2010).

#### Tools

We found that CCS programs that afford access to scientific tools open up opportunities for youth to practice multiple types of participation. This finding is in line with the studies by Dohn (2011), Palmer (2004), Roesch, Nerb, and Riess (2015), and Schwartz, Thomas-Hilburn, and Haverland (2011). Tools and instructions on how to use them shape the pattern of participation and the manner in which tools are used by participants, and this, in turn, may affect the quality of expected outcomes (Gedera and Williams 2015). Youth participation is mediated by tools, as they transform the objective into either desired or unexpected outcomes (Plakitsi 2011). Our study showed that, among the three programs, access to scientific tools and understanding how to implement them in the field (mediated by the facilitator/community) varied, and influenced the ways youth participated. Further, the programs that offer immersion in authentic experiences, access to scientific tools, and performing different types of participation multiple times lead to skill-building, enable participants to gain mastery, and contribute to solving real-life problems (Ballard, Dixon, and Harris 2017a). For example, SP youth developed expertise in using tools and gained ownership over taking better photos and submitting data, and some SAC participants took ownership over tools and taught other youth how to use them. Having such experiences may lead participants to move from peripheral participation to full participation (Lave and Wenger 1991).

#### Community

Interaction with others in CCS settings, from peers to facilitators, supports and opens up opportunities for different types of youth participation, specifically when youth could receive or provide feedback. Within the environmental CCS activity systems, youth participation most often occurs in collaboration with others with whom they may share their knowledge. In these situations, youth go beyond the individual level to communicate their knowledge with the community on a collective level (Roth, Lee, and Hsu 2009). Facilitators communicate the objective and mediate the division of labor by introducing the tools, the instructions for using the tools, and connecting youth actions to the broader objective (Roth, Lee, and Hsu 2009; Heo and Lee 2013). The interaction of some SP youth with the scientific community (i.e. NHM researchers) throughout the program made them more motivated to use the practices they learned during participation. This leads us to conclude that the motivation of participants to be engaged in an environmental program and become competent in the use of specific tools to Record data was bound up in the environmental paradigm of the community to which the participants belong (Roth and Lee 2007). For our programs, this paradigm was that biodiversity discoveries can be made anywhere, including in urbanized areas. Roth and Lee (2007) found similar outcomes and referred to youth role and performance within a bigger environmentally-focused community as "fibers in a strand (197)." The efforts of these participants can be seen as a form of "legitimate peripheral participation" because participants take part in legitimate activities of that community (Lave and Wenger 1991). Further, these findings may have implications for thinking about CCS projects as a "community of practice" (Merenlender et al. 2016) though the ways in which this might be true for youth-focused CCS programs deserve further research.

#### Division of labor

To have a successful activity, according to Engeström (2000, 2010), each program must contain multiple options for roles or divisions of labor for all participants at different stages of their engagement, plus transparent and clear communication about what each role contains and who conducts which task. In a successful program with a clear CCS objective, there are multiple roles for individuals to try. In our study, SP participants tried a variety of roles including explorer, observer, identifier, documenter, and recorder. In SAC, youth division of labor correlated with their access to tools, in that youth who practiced with the bug net frequently began to identify their role as "bug catcher", and those who practiced with the magnifying containers began to identify as the "bug observer". In BSS, having few activities with a CCS objective limited youth CCS participation and taking on CCS roles, though they may have explored other non-CCS roles outside the focus of the study. This is aligned with the study by Pitt and Schultz (2018), which found that when the program objectives are clear to youth, it positively affects their engagement and understanding of their role in the monitoring effort. Kaptelinin and Nardi (2012) suggest that this division of labor and the accessibility of tools are interconnected because access to scientific tools and participants' specialization in using them creates and reinforces the division of labor. Participants took ownership of the tool over time, and developed expertise in using the tools to find wildlife, document observations, and submit biological records (Dohn 2011; Palmer 2004; Roesch, Nerb, and Riess 2015; Schwartz, Thomas-Hilburn, and Haverland 2011).

### Changes through time

Youth participation in multiple sessions helped us to understand changes in their engagement through time (Brown, Collins, and Duguid 1989; Nasir 2005; Rogoff 1995; Parrish et al. 2019). For youth who did not show change over time (*n* = 11), the limited opportunities to participate in the program and engage with CCS practices may have impacted their opportunity to try and/or deepen different types of participation over time. These groups were mostly engaged in activities with environmental education objectives such as building sandcastles in BSS and drawing an insect in SAC and therefore had fewer opportunities to explore nature using inquiry-based science practices. This limited their opportunities to experience CCS-based division of labor and use science tools (Lewis and O'Brien 2012). Multiple sessions of participation in CCS provided the opportunities for youth to try the same type of participation multiple times (i.e. *intensification),* or to try different types of participation (i.e. *diversification).* Taking on new roles, gaining mastery, and feeling competent in performing disciplinary practices may positively affect youth identity development (NRC 2009; He et al. 2019). Repeated participation and interaction with scientific tools and a broader community in the CCS programs enabled participants to develop a "habit" (Hatton et al. 2019) by practicing participation in different ways, trying different divisions of labor, and gaining mastery of tools and expertise in certain types of participation. This is consistent with previous studies that investigated the impact of multi-day experiences in informal settings on youth learning (Fields 2009; Gibson and Chase 2002; Plakitsi 2011, Parrish et al. 2019). Through repeated participation in CCS, participants developed scientific practice, gained specialized content knowledge and expertise, and had multiple opportunities to participate in CCS practices. Understanding youth participation and how it changes through time matters in understanding how youth act in the context of CCS both individually and in interaction with others (Krasny and Roth 2010). Further, understanding youth participation may help inform future CCS program design as we discuss below.

### Implications and recommendations for CCS program design for environmental education

**Recommendations for impactful ongoing CCS youth programs:**

- **Project Design**
- Create a clear program objective and at the beginning of the program build in time to explain this intention clearly. Repeatedly restate this objective throughout the program.
- Design activities that build skills, tool mastery, and allow youth to take on appropriate roles for each type of participation. If a program's goal is for youth to take part in all aspects of CCS, Exploring through Recording, recognize that Recording and Documenting are less frequently observed in youth and greater assistance may be required to help youth develop these skills. (That is, design for diversification).
- Design activities that reinforce the program objective and build in repetition of activities to allow youth to develop competency and take on new roles through time. (That is, design for intensification).
- Build in opportunities for community interactions for youth with peers, facilitators, parents/guardians, and scientists. This inspires and supports youth to communicate about their observations, share and Record their findings, and receive feedback, all of which reinforce other activities.
- (If delivering CCS activities as part of a wider environmental education program) place a strong emphasis on youth CCS participation and their contribution to science. This will differentiate CCS from the other activities.
- **Training Facilitators**
- Ensure facilitators can clearly state the program's objective, and understand the importance of repeating the goal to enhance youth learning
- Ensure facilitators understand the importance of tools, community, and division of labor.
- **Access to Tools**
- Provide necessary training for facilitators about the use of scientific tools, and how youth can appropriately use these tools. This can allow youth to take on new roles and develop skill mastery which may support identity development.
- **Scaling and the degrees of separation between the youth and the CCS practitioners who design the program**
- Ensure program design includes close ties to, and opportunities to connect with the CCS practitioners to support youth participation in all steps of CCS.
- Consider the scale of the program. If the hope is to design a program that will scale, environmental education components tend to scale more easily than CCS components. Increasing the scale brings a higher degree of separation between CCS project designers or lead scientists and the youth, and therefore a greater amount of training and support is needed for program deliverers to lead the CCS components.

## Conclusions

Overall, our use of Activity Theory as a lens for examining youth participation in CCS allowed us to identify key components of the activity systems of ongoing CCS programs. Our findings can inform the design of environmental CCS programs, particularly how the program features could promote and constrain opportunities for youth participation and experiences with environmental science. We conclude that communicating a clear CCS objective of the program and aligning the goals of all the associated activities with the main objective can help youth gain awareness of their role, understand the impact of their individual action, and how their participation could contribute to the research effort. Further, access to a variety of science tools and repeated use of the same tool led participants to try different roles within CCS and gain mastery of science disciplinary skills over time. Facilitators have a key role in communicating the scientific research and monitoring objectives of the CCS program, and training youth to use and practice repeatedly with scientific tools. By offering multiple entry points for participating in the scientific process within the biodiversity-focused CCS programs, well-designed ongoing CCS programs can provide opportunities for youth to explore and take up a variety of roles in environmental science research and monitoring. This extension and deepening of participation across multiple roles speaks to the CCS practitioner community's goals of moving participation beyond simple, transitional data collection/submission and towards activities which mirror the multi-role nature of professional scientists and their research practices. This opens up youth to developing a greater personal stake in the research outcomes of the project specifically and in science generally-all in a world where critical and scientific thinking are ever more vital.

## Supplementary Material

Appendix

## Figures and Tables

**Table 1:** Overview of each CCS program, program objective and structure, participants, and site characteristics.

	Science Action Club (SAC )	SuperProject (SP)	Big Seaweed Search (BSS)
**Natural history museum**	California Academy of Sciences (CAS ), U.S.	Natural History Museum of Los Angeles County (NHMLAC ), U.S.	Natural History Museum, London (NHM London), UK
**Scientific research or monitoring focus**	For this research project we studied one of the three units offered by NHM London: “Bug Safari” unit which focuses on understanding biodiversity through collection of arthropod species-occurrence data.	Assessing urban biodiversity, through regular nature surveys - specific focus on species occurrence data (i.e. snails, slugs, reptiles, amphibians, and mammals) - at neighborhood sites in the Greater Los Angeles Area, California.	Monitoring seaweed distribution and abundance through collection of presence-absence data, and description of coastal features around the UK.
**Participant involvement in data collection according to CCS program design**	Youth in afterschool programs across the U.S. follow a prescribed data collection protocol to search for, observe, and sometimes take photos of arthropods to be uploaded onto the iNaturalist platform via the app or website.	Families across the Los Angeles area follow prescribed data collection protocols to search their backyards and neighborhoods for any organisms, observe, and take photos of organisms and upload onto the iNaturalist platform via the app or website and fill in survey reports on a project website.	Participants (adults and youth across the UK) follow a prescribed data collection protocol to search for, photograph, and identify seaweed. Data are documented on paper datasheets and then uploaded with photos to the BSS website.
**Biological data end-user(s)**	“Research grade” observations on iNaturalist (agreed upon by two-thirds of identifiers on iNaturalist) are added to the Global Biodiversity Information Facility (GBIF).	Museum research staff use observations which contribute to five ongoing urban biodiversity research surveys in L.A. “Research grade” observations on iNaturalist are added to the Global Biodiversity Information Facility (GBIF).	Museum staff, scientific researchers, and observations contribute to ongoing environmental/climate change research.
**Relationship between CCS program leadership and ‘on the ground’ program delivery**	CAS education staff designed SAC curriculum and educator training, and worked with a CAS entomologist to design the “Bug Safari” unit and CCS protocols. Afterschool educators implement curriculum, including supervising youth participation in CCS activities.	NHMLAC education and science staff co-designed CCS protocols and program activities, and trained families in CCS data collection activities. Parent/guardian(s) supervised youth participation in CCS data collection activities.	NHM London designed BSS protocols and provided written instructions and supporting materials for participants. No other training was provided. Participants may carry out the survey as an individual or group. In our case study, a youth group was led by a local wildlife charity, who embedded BSS within a wider Environmental Education program. Environmental Educators with the support of teachers supervised youth participation in CCS data collection activities.
**CCS program delivery at our case study sites**	CAS museum staff train regional SAC trainers on the SAC “Bug Safari” unit curriculum through self-paced online and in-person training. The SAC trainers in turn train activity leaders who also participate in the online and/or in person training prior to leading programming over 12 environmental education lessons including 8 CCS components in which youth collect data about arthropods; youth or facilitators upload arthropod data to an online database.	NHMLAC educators and scientists train parent/guardian(s) and youth at the beginning of the project on CCS practices. Youth, with or without direct supervision from parent/guardian(s), collect data during at least two sessions each month (at a home site, a local neighborhood site (e.g. local streetscape), and an open space site (e.g. local park). Youth or parent/guardian(s) upload data to iNaturalist and to an online database. Youth and families are invited to participate in community events with special presentations or tours given by museum scientists and educators quarterly and attend a larger event at the halfway point and end of the program.	A local wildlife charity delivers a coastal Environmental Education Program for youth. The educators included BSS as a CCS component in a wider program, but without specific CCS -related training. Youth were a school class on an environmental education field trip comprising four beach activity sessions including environmental education activities and CCS data collection. Adult facilitator(s) collect CCS data sheets and submit data online.
**Participant audience for overall CCS program and at our case study sites**	Overall: Middle school youth (ages 10–14). Case Study Sites: Elementary and Middle school youth (ages 7–14).	Overall: All ages Case Study Sites: Youth participation is most supported for kindergarten through high school youth (ages 4–19).	Overall: All ages Case Study Sites: Elementary school youth (ages 7–10).
**Location of activities at case study sites**	Schoolyards and community centers in California within approximately 1 h drive from CAS in the San Francisco Bay Area, California.	Family homes (backyards and apartment complex common areas), public gardens and parks, and open green spaces in San Fernando Valley and South Los Angeles areas, California.	Rocky shore and single beach coastal sites in Sussex, UK.
**Educational program duration and frequency of participation for overall CCS program and at our study sites**	Overall: 5–10 weeks Case Study Sites: 12 h in total, across 12 weekly sessions. March – May 2018	Overall: 1 year Case Study Sites: Approx. 36–54 h in total, across 24–36 bi-monthly sessions. April 2017 – July 2018	Overall: 1 day–1 week Case Study Sites: 15+ hours in total, across 4 consecutive weekly sessions. June – July 2018

**Table 2:** Summary of case study data collection.

Case	Total no. youth participants	No. focal youth (male and female*)	Age* group of focal youth**	No. individual youth observations	No. sessions observed
SAC (3 sites)	~30	9 (7 M, 2 F)	Middle school	28	14
SP	6	5 (4 M, 1 F)	2 Elementary & 3 High school	19	19
BSS	31	5 (3 M, 2 F)	Elementary	17	4
**Total: 5**	**~67**	**19 (14 M, 5 F)**	**19**	***65***	**37**

* All demographic information including the age and gender were suppositions and were not confirmed by participants during our observation due to ethics approval.

** elementary (5–10 yrs), middle school (11–13 yrs), high school (14–19 yrs)

~ Participant numbers varied per session and the estimate reflects the average number of participants per session.

**Table 3:** CCS -related types of participation in biodiversity recording projects (from Lorke et al. 2021).

	Definition
**Types of participation in community and citizen science**	
Exploration	Exploring nature to discover organisms, searching for wildlife
Observing	Using one's senses to find and study organisms
Identifying	Identifying, in the sense of naming which organism was observed
Documenting	Documenting the observations by generating evidence (e.g. a photograph or text)
Recording	Recording, in the sense of making the documented observation available for biodiversity monitoring or research purposes (e.g. uploading to iNaturalist)

**Table 4:** Activity system components and the definitions for NHM-led CCS contexts.

CHAT Components	Definition in CCS Context (i.e. Setting Features)
Subject	Individual participant in the CCS program
Object (Objective)	The stated goals of the activity. Based on conversations and artifacts, the reasons that the program designers and facilitators use to explain why this program is taking place.
Tools	Physical resources that participants can use in the learning setting to accomplish the activities (e.g. magnifying glasses, insect nets, or plastic spoons to isolate and manipulate small organisms).
Rules	The expectations and norms within the activity space.
Division of Labor	Information about who has been assigned/ or taken on different tasks and what those tasks are.
Community	The people including peers, parents, facilitators, siblings, etc. who are present during youth participation and the implementation of the program and may support and mediate youth engagement in the activity, and applying tools.
Outcomes	Whether participants meet the objective of the activity

**Table 5:** Indicators and examples from youth profiles showcasing different types of participation in three NHM-led ongoing CCS programs.

Type of participation in CCS across three programs (Total participants 8 SAC, 5 SP & 3 BSS)	Indicators of the type of participation in CCS			Example
	SAC	SP	BSS			
**Exploring/Discovering** (*No. of participants: 5 SAC, 5 SP, & 3 BSS*) Youth engaged in searching for wildlife in a habitat (e.g. looking under logs, turning over rocks), with or without tools, independently or guided by others (e.g. a facilitator or another youth).	• Exploring the green space outside of the after-school club • Looking for insects • Using tools including bug net, beating sheet, and pooter to catch insects • Looking under rocks to search for insects	• Exploring different habitats including backyard, park, neighborhood • Looking for organisms including plants, insects, and birds • Revisiting the same location over time to check on previously observed organisms • Looking for certain species in a certain habitat • Using bug net to capture invertebrates	• Exploring the beach • Looking for natural elements including shells, shark egg cases, and seaweed	*During the first ”Bug Safari“, Sophie is walking slowly around with the bug net: ”I want to catch a bug“. Her friend shows her to look in the tree where the spiders are. Sophie looks at the bark on a nearby tree and half heartedly waves the bug net, looks inside and exclaims, ”we actually caught something!“ Sophie asks her to help her transfer the bug to the Petri dish. (Middle school female participant, SAC, ”Bug Safari“, First observation)*
**Observing** (*No. of participants: 4 SAC, 5 SP, & 3 BSS*) Youth used their senses to find and watch wildlife, with or without tools.	• Looking closely at insects’ behavior • Looking in a Petri dish to watch an insect closely • Looking at the insects and their features closely in the outdoor greenspace	• Looking closely at the species including spiders, bees, or spider’s webs • Watching a wasp closely and listening to the buzzing sounds • Using sense of touch and smell to identify species • Looking closely at the evidence of a certain species such as eggs of a spider or trail of a snail	• Walking on the beach and looking at different features or seaweed • Looking at a very large rock and a crevice between the rocks • Observing organisms in a tidepool • Observing closely the shark egg cases collected by the facilitator • Observing seaweed and tasting it	*--Mason finds and spends time looking at seaweed at the beach. He starts pressing the ‘bladders’ on a piece of seaweed, squeezing them down against the bottom of the bucket. Then suddenly he takes his hands out and licks his fingers. (Elementary school male participant, BSS, Second observation)*
**Identifying** (*No. of participants: 3 SAC, 5 SP, & 1 BSS*) Youth established or suggested the identification of an organism, referring to a species name, common name, or other taxon name. Identification included different sub-categories, such as: ’Identification via iNaturalist’ (youth use the built-in AI feature to identify an organism); ’Identification according to prior knowledge’ (youth name a species or taxonomic group that is familiar to them); and ’Identification according to features’ (youth refer to the shape, color or pattern of the organism).	• Using iNaturalist (mostly with facilitators) to identify insects • Using basic knowledge of insect characteristics to differentiate from spiders • Using prior knowledge to identify species	• Using evidence (e.g. feature or holes in the ground) to identify a species • Using iNaturalist (mostly youth and sometimes parents) to identify species • Using prior knowledge to identify • Getting feedback from adults (parents or research observers) to identify species • Using observation skills to identify a species such as touching fennel and smelling it	• Using physical characteristics and identification sheet to identify seaweed and shark eggs	*--Charlie squats down in front of a manicured grass lawn and he points out to the observer a plant that is the same size as the rest of the grass, but a different texture and darker green. He tells the observer it is fennel, and he pulls it out a little bit for the observer to look at the leaves. He tells the observer to touch it and then to smell her fingers. (High school male participant, SP, Third observation)*
**Documenting** (*No. of participants: 6 SAC, 5 SP, & 0 BSS*) Youth produced some form of artifact that documented an observation including taking a photo, writing down a location, collecting the organism, using equipment (e.g. vial or net), or drawing the organism. Any documentation has the potential to be shared for monitoring and/or research purposes, but it is not necessary for this step.	• Taking photos of insects with iNaturalist (sometimes using magnifying lens too), mostly by facilitator while youth is aware or is part of the process • Taking photos of the insect inside a Petri dish	• Taking photos of different species by camera or iNaturalist app, by youth or by others (parents, research observer) while youth suggested or is aware • Taking pictures of evidence of a species (e.g. animal scat) to ID the animal • Taking photos from multiple angles to make a clearer observation	• Filling out a data sheet at the beach to document the species observed	*Ashley takes her phone out and starts taking photos of all the bees on the flowers. She gets really close to the bees for multiple photos she snaps. (High school female participants, SP, First observation)*
**Recording** (*No. of participants: 1 SAC, 4 SP, & 0 BSS*) Recording was captured when one or more biological records were created and submitted in any form that could then be used by scientists, or by a wider community, for research or biodiversity monitoring purposes. Recording was carried out by youth, or by others (facilitator, peers, or other adults) with youth involved and/or that data were being recorded.	• Submitting photographs and associated data to iNaturalist by youth or facilitator	• Submitting photographs and associated data to iNaturalist by youth, parents, or research observer	• Handing completed paper recording forms to identify shark eggs to a facilitator or sending them to museum staff	*Laurence comes across a whole bunch of acorn nuts on the sidewalk and tells the observer ”This tells us that there is a squirrel nearby.“ Then he starts looking in the trees for a squirrel. He finds one in the trees and tries to get his mom to take a photo of it, but every time they get closer the squirrel goes to the other side of the tree making it very hard. He decides to go back to the nuts and take pictures of them instead, to upload them to iNaturalist. (Elementary male youth participant, SP, Second observation).*

**Table 6:** Youth engagement in CCS -related types of participation in three CCS programs (black andwhite colors represent presence and absence, respectively.).

Program	CCS Programs & Focal Youth														
				SAC						SP				BSS	
Type of participation	Conor	Joey	Samuel	Oliver	Sophie	Sebastian	Zara	Dan	Ashley	Laurence	Scott	Charlie	Renee	Gabriel	Mason
**Exploring**															
**Observing**															
**Identifying**															
**Documenting**															
**Recording**															
**Total**	5	4	3	2	2	2	1	5	5	5	4	4	2	2	2
