# Supplementary material for: Examining youth participation in ongoing community and citizen science programs in 3 different out-of-school settings
Source: Environ Educ Res. Author manuscript; Available in PMC 2022 Dec 2. (PMC7613686; doi:10.1080/13504622.2022.2078480)
Supplement: Appendix [file EMS146400-supplement-Appendix.pdf]

Appendix A. Co-occurrence of the CCS-related types of participation and the components of the second generation of CHAT. The darkness of the shading (legend) represents the relative frequency of the co-occurrence. The numbers within each cell are the number of focal youth whose profile included the pairing of the type of participation and CHAT components

No. *of co-occurred episodes*	*Color code*
0–9	
10–19	
20–29	
>29	

	Second Generation of CHAT Components				
	Community	Object	Rules	Tools	Division of labor
**CCS related Type of Participation**					
Exploration & Discovery	**11**	4	3	**13**	**6**
Observing	**11**	3	3	**11**	**8**
Identifying Organisms	**5**	1	0	**7**	**1**
Documenting & Reporting	**9**	2	2	**11**	**9**
Recording	**3**	0	3	**4**	**1**
